# Chatbot-Delivered Online Intervention to Promote Seasonal Influenza Vaccination During the COVID-19 Pandemic: A Randomized Clinical Trial

**DOI:** 10.1001/jamanetworkopen.2023.32568

**Published:** 2023-09-11

**Authors:** Zixin Wang, Paul Shing-fong Chan, Yuan Fang, Fuk-yuen Yu, Danhua Ye, Qingpeng Zhang, Martin C. S. Wong, Phoenix K. H. Mo

**Affiliations:** 1 JC School of Public Health and Primary Care, The Chinese University of Hong Kong, Hong Kong SAR, China; 2 Department of Health and Physical Education, The Education University of Hong Kong, Hong Kong SAR, China; 3 Musketeers Foundation Institute of Data Science, The University of Hong Kong, Hong Kong SAR, China; 4 Department of Pharmacology and Pharmacy, LKS Faculty of Medicine, The University of Hong Kong, Hong Kong SAR, China

## Abstract

**Importance:**

Receiving seasonal influenza vaccination (SIV) is important for adults during the COVID-19 pandemic. There are few robust evaluations of tailored interventions for improving SIV uptake among adults 65 years or older.

**Objective:**

To evaluate the relative efficacy of a stages of change (SOC)-tailored online intervention compared with a standard, non–SOC-tailored online intervention in increasing SIV uptake among Hong Kong residents 65 years or older.

**Design, Setting, and Participants:**

This nonblinded parallel-group randomized clinical trial was conducted between December 1, 2021, and July 31, 2022, in Hong Kong, China. Eligible participants were 65 years or older, had Cantonese- and/or Mandarin-speaking skills, were community-dwelling, had Hong Kong residency, were smartphone users, and had not received SIV for the 2021 to 2022 influenza season. Participants were recruited through random telephone calls, and those who completed the baseline telephone survey were randomized to either the intervention or control group. Both complete case and intention-to-treat (ITT) analyses were performed.

**Intervention:**

In the intervention group, a simplified rule-based chatbot first assessed participants’ SOC related to SIV uptake and then automatically selected and sent participants SOC-tailored online health promotion messages (videos) through a messaging application (WhatsApp; Meta) once every 2 weeks for 4 sessions. In the control group, the chatbot sent a link to access through the messaging application a standard online health promotion message (video) covering general SIV information every 2 weeks for 4 sessions.

**Main Outcomes and Measures:**

The primary outcome was self-reported SIV uptake at month 6, which was validated by the research team. The secondary outcome was SOC measured at both baseline and month 6 by validated questions.

**Results:**

A total of 396 participants (mean [SD] age of 70.2 [4.3] years; 249 females [62.9%]) were randomized to the intervention (n = 198) or control (n = 198) group. The ITT analysis showed that the validated SIV uptake rate was higher in the intervention group than the control group at month 6 (50.5% vs 35.3%; *P* = .002). The mean (SD) SOC score was higher in the intervention group than the control group (2.8 [1.4] vs 2.4 [1.4]; *P* = .02). More participants in the intervention group completed at least 1 episode of intervention than in the control group (77.3% vs 62.6%; *P* < .001).

**Conclusions:**

Results of this trial indicate that the SOC-tailored online intervention was more effective than the non–SOC-tailored intervention and may be a sustainable new method in increasing SIV uptake among adults 65 years or older.

**Trial Registration:**

ClinicalTrials.gov Identifier: NCT05155241

## Key Points

**Question:**

Is an online intervention tailored to the stages of change (SOC) more effective than a non–SOC-tailored intervention in increasing seasonal influenza vaccination (SIV) uptake among adults 65 years or older?

**Findings:**

In this randomized clinical trial of 396 Hong Kong residents 65 years or older, those who received SOC-tailored online intervention every 2 weeks for 4 sessions significantly increased SIV uptake compared with those who received non–SOC-tailored intervention once every 2 weeks for 4 sessions.

**Meaning:**

Findings of this trial indicate that SOC-tailored intervention may be an effective and a sustainable new method for increasing SIV among adults 65 years or older.

## Teaser

This randomized clinical trial examines a technology-based promotion of free seasonal influenza information and vaccination for Hong Kong residents 65 years or older.

## Introduction

Seasonal influenza vaccination (SIV) is effective in preventing influenza and all-cause mortality among individuals 65 years or older.^1^ In line with the World Health Organization, the Hong Kong Centre for Health Protection recommends all local residents 65 years or older to receive SIV once every year.^2^ The Hong Kong government offers free SIV to residents of this age group.^3^ However, the SIV coverage among these individuals remains inadequate, with 44.7% in 2020 to 2021 and 40.4% in 2021 to 2022.^4^ There is an urgent need to improve SIV uptake among adults 65 years or older.

There were a few randomized clinical trials (RCTs) comparing the mailing of simple and standard reminders vs no intervention; however, the effect size of these interventions was relatively small.^5^ Although more intensive interventions (eg, telephone education sessions or home visits) were more likely to be effective and had a larger effect size,^5^ these interventions were resource-demanding. In Hong Kong, the government disseminates standard information about SIV through mass media channels. Only 1 RCT was conducted in Hong Kong, and it found that a standard one-to-one, face-to-face verbal education by medical students was effective in increasing SIV uptake compared with no intervention (33.6% vs 25%; *P* < .05).^6^ Although tailored informational interventions are more effective than standard informational interventions for facilitating behavioral changes,^7^ there was a lack of tailored interventions promoting SIV among adults 65 years or older.

The Transtheoretical Model (TTM)^8^ was used to guide the development of health promotion in this trial. Stage of change (SOC) is the core of TTM and is a measure of readiness for behavioral change.^9^ According to the TTM, different health promotion strategies should be applied to people at different SOC. A meta-analysis showed that interventions tailored to current SOC were more effective than non–SOC-tailored interventions, especially among less motivated individuals.^9,10^ To our knowledge, no study has applied SOC-tailored intervention based on the TTM to promote SIV.

A chatbot is a computerized program that acts to replicate human interaction through text, speech, and visual forms of communication.^11^ The chatbot is potentially useful to deliver SOC-tailored intervention promoting SIV. A recent systematic review demonstrated high efficacy of chatbots in promoting healthy lifestyles, smoking cessation, and treatment adherence and addressing issues related to substance misuse.^12^ A few pilot studies have applied chatbots to provide information and answer questions about COVID-19 vaccines and have observed substantial increases in acceptance and intention to receive COVID-19 vaccines among users.^13,14,15^

This RCT was conducted to evaluate the relative efficacy of a SOC-tailored online intervention (the intervention group) compared with a standard, non–SOC-tailored online intervention (the control group) in increasing SIV uptake among Hong Kong residents 65 years or older. The online interventions in both groups were delivered by a simplified rule-based chatbot. We hypothesized that the intervention group would have higher absolute SIV uptake during the study period compared with the control group.

## Methods

### Study Design and Participants

**Figure 1.**
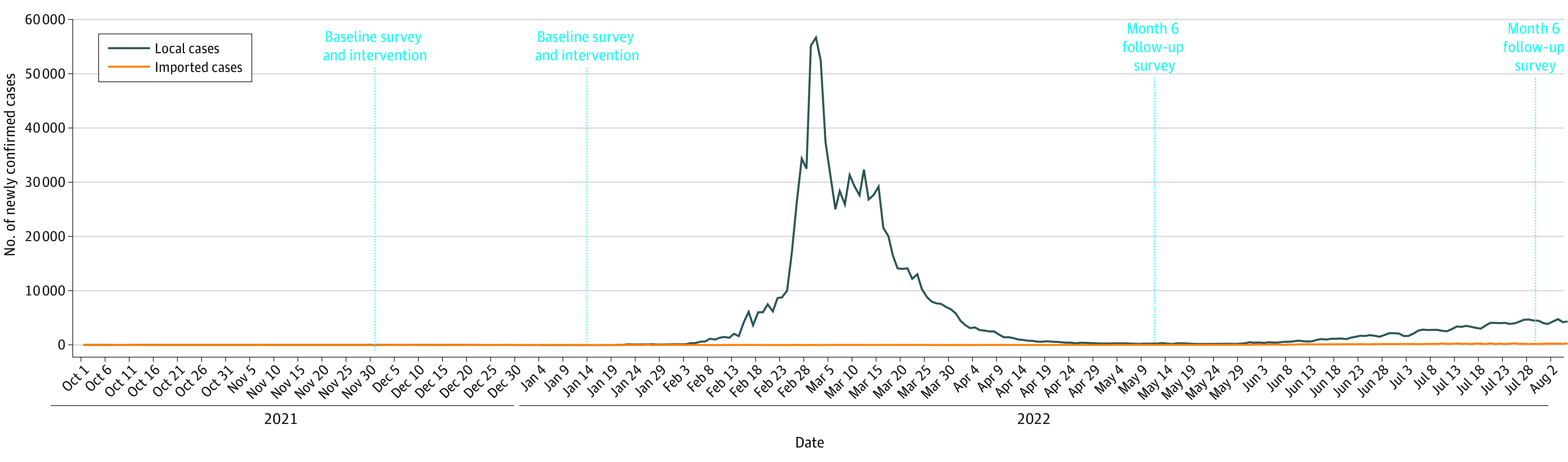
COVID-19 Situation During the Study Period in Hong Kong

A nonblinded 2-group parallel RCT was conducted between December 1, 2021, and July 31, 2022, in Hong Kong. The COVID-19 situation in Hong Kong during the study period is shown in Figure 1. The trial protocol and statistical plan are available in Supplement 1. The Survey and Behavioral Research Ethics Committee of the Chinese University of Hong Kong and the joint CUHK-NTEC Clinical Research Ethics Committee approved the study. All participants provided verbal informed consent. We followed the Consolidated Standards of Reporting Trials (CONSORT) reporting guideline.

Inclusion criteria were age 65 years or older, possession of a Hong Kong identity card, ability to speak Cantonese and/or Mandarin, willingness to participate in follow-up by telephone, access to a smartphone, and no SIV uptake for the 2021 to 2022 influenza season. Exclusion criteria included cognitive impairment, blindness or deafness, inability to communicate effectively, and known contradictions to SIV as indicated by the Centre for Health Protection.^16^ A previous study showed that 55.7% of Hong Kong adults 65 years or older intended to receive SIV in the next year during the COVID-19 pandemic.^16^ For planning purposes, we conservatively assumed that 50% of those in the control group would show an intention, and 40% of those with such an intention would obtain SIV (20% of the control group).^17^ We used a smallest detectable difference of 15% between the intervention group and the control group (35% in the intervention group). Therefore, we needed 138 per group to achieve planned effect sizes and a power of 0.8 and α = .05. Assuming the dropout rate to be 30% at month 6, 198 participants per group was required (PASS 11.0; NCSS).

Participants were recruited through random telephone sampling. All household telephone numbers listed in the most up-to-date telephone directories (approximately 350 000) were input into a spreadsheet file (Excel; Microsoft Corp). The function of select random cells generated 4000 household numbers. Trained interviewers conducted the telephone calls from 6 to 10 pm on weekdays and from 2 to 9 pm on Saturdays to avoid undersampling of working individuals. If no one in the household answered the initial call, 4 more follow-up calls were made on different days and hours before the household was considered to be nonvalid. If there was more than 1 person in the household who was 65 years or older, the person whose most recent birthday was closest to the interview date was invited to join the study. Interviewers screened prospective participants for eligibility, briefed them about the study, and guaranteed their anonymity and right to quit at any time. These individuals were informed of the available hotline for inquiry during office hours. Since there was no face-to-face contact, verbal informed consent was obtained. The interviewers signed a form pledging that the participants had been fully informed about the study. A supermarket coupon worth HK$50 (US $6.40) was mailed to the participant after completing the telephone surveys at baseline and 6 months after completing the interventions (month 6).

### Randomization, Masking, and Development of the Intervention

At the end of the baseline survey, the interviewers connected participants to WhatsApp, a messaging application (Meta), used for the intervention system. Participants were then randomized evenly to either the intervention group or the control group by a randomization algorithm that was built into the intervention system. The automated randomization occurred online, and the randomized group was concealed from the research team.

Details of the intervention development are shown in eAppendix 1 in Supplement 2. The architecture of the intervention system used in this trial was adapted from a mature rule-based chatbot for smoking cessation.^18^ To meet the needs of adults 65 years or older, we simplified the human-machine interactions and used videos instead of chats to deliver health promotion messages. The chatbot was integrated with the messaging application through its public web API (application programming interface) services. Details of the simplified rule-based chatbot are provided in eAppendix 2 in Supplement 2.

### Intervention and Control Groups

Participants in the intervention group watched through the messaging application 1 of the online health promotion videos (lasting approximately 3 minutes) tailored to their SOC regarding SIV uptake once every 2 weeks for 4 sessions at week 0, 2, 4, and 6. The health communication messages in these videos followed the strategies to facilitate progression in SOC recommended by the TTM.^13^ To prevent participants from watching the same video twice, we prepared 4 slightly different versions of online videos corresponding to each SOC. In each session, the intervention system randomly selected 1 version for the participants.

**Figure 2.**
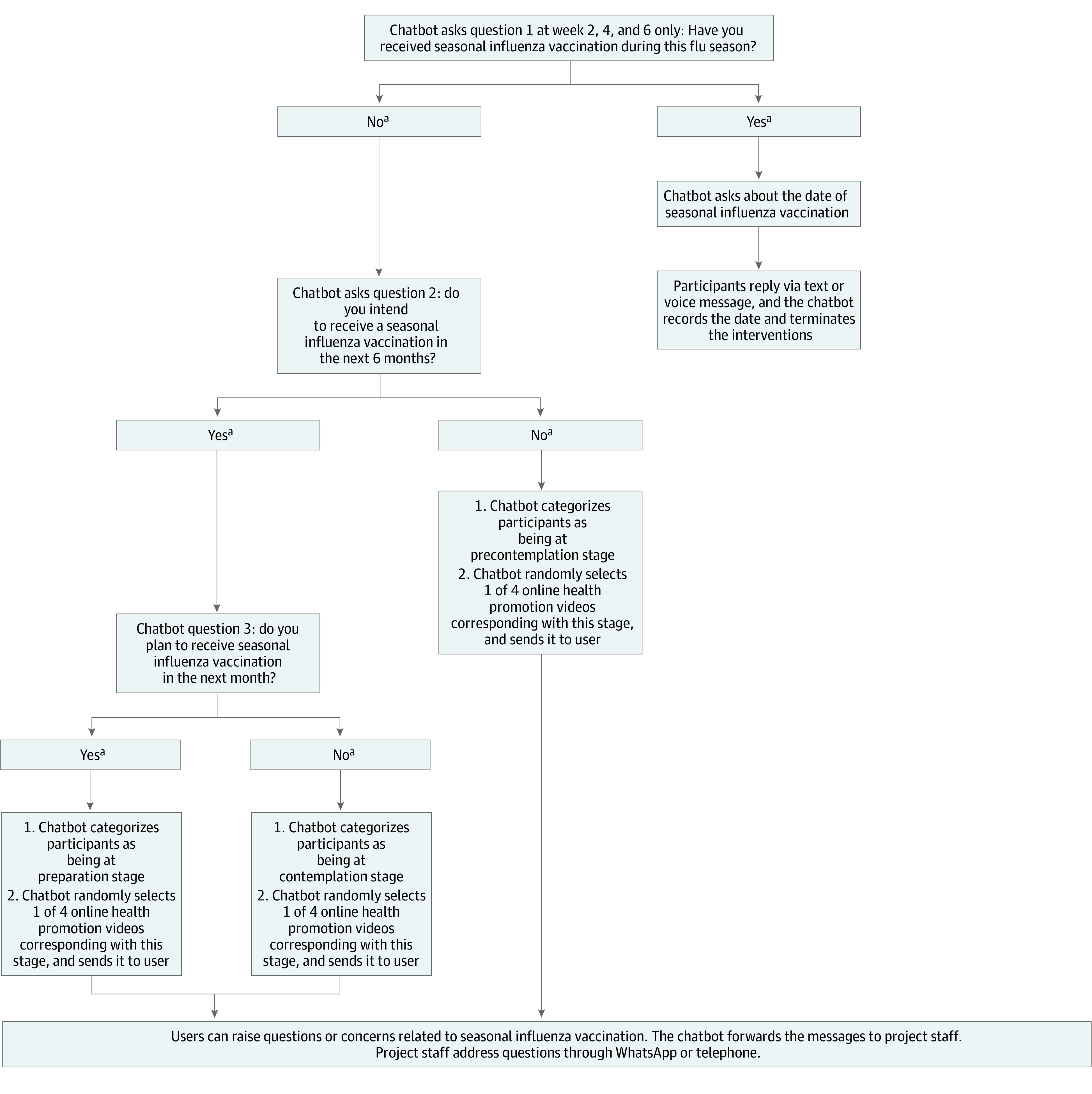
Workflow of the Intervention System ^a^Participants could click a button on screen, input a number or letter representing an answer (1 or Y = yes; 2 or N = no) or use voice messages to answer the questions.

At the beginning of each session, the intervention system assessed participants’ SOC by asking them 2 questions: whether they intended to obtain SIV in the next 6 months, and whether they planned to do so in the next month. Stages of change were as follows: precontemplation stage, defined as without intention to obtain SIV in the next 6 months; contemplation stage, defined as intention to obtain SIV in the next 6 months but without plans to do so in the next month; and preparation stage, defined as intention to obtain SIV in the next month. The same measurements and definitions of SOC were commonly used in studies of vaccination behaviors.^19,20^ Starting from the second session, the intervention system asked participants an additional question of whether they had already received SIV for the upcoming influenza season (yes or no). If participants selected yes, they were considered to be at the action stage. The intervention system made a record and then terminated the program automatically. The workflow of the intervention system is shown in Figure 2. Scores for each SOC were as follows: 1 for precontemplation stage; 2 for contemplation stage; 3 for preparation stage; and 4 for action stage, with the highest scores indicating greatest readiness level.

For participants at the precontemplation stage, the online video aimed to increase their awareness about the importance of SIV uptake. The video was presented by a primary care physician, who covered the following topics: (1) high risk of influenza infection and severe consequences among adults 65 years or older, (2) increased risk of death associated with influenza and COVID-19 coinfection, (3) SIV as an effective means of protecting adults 65 years or older from seasonal influenza, and (4) free SIV available for adults 65 years or older. These individuals can also receive subsidized SIV at private clinics.

For participants at the contemplation stage, the online video aimed to increase perceived advantages, increase perceived self-efficacy (defined as a person’s confidence in receiving SIV), and reduce perceived disadvantages related to SIV uptake. In the video, the same primary care physician discussed the efficacies of SIV in preventing seasonal influenza and reducing related hospitalization and deaths as well as explained that the common adverse effects of SIV were mild and severe adverse effects were rare. Testimonials of vaccinated adults 65 years or older regarding adverse effects were also presented. At the end of the video, the primary care physician gave recommendations and encouraged these adults to make plans to receive SIV before the upcoming influenza season.

For participants at the preparation stage, the online intervention aimed to assist with developing and implementing their concrete action plan and increasing their perceived self-efficacy related to SIV uptake. The video provided the location and contact information of facilities in different districts offering free SIV for individuals 65 years or older and explained the procedures for making an appointment. Additionally, participants were asked to click a box to indicate when and where they planned to obtain the SIV.

For participants in the control group, the intervention system automatically sent a link through the messaging application to access a standard online video (lasting 2 minutes), which covered basic information about who could, when to, and where to receive the SIV, once every 2 weeks for 4 sessions at weeks 0, 2, 4, and 6. Such information was identical to that disseminated through mass media channels by the Hong Kong government.

### Study Outcomes

Self-reported uptake of SIV measured at month 6 was the primary outcome. This outcome was validated by requesting participants to upload an image of their SIV receipt, blocking personal identification, via the messaging application account used in this trial. No incentive was offered for validation.

The secondary outcome was SOC measured at both baseline and month 6. Measurements and definitions of SOC were the same as those used by the intervention system. At each intervention session of participants in the intervention group, the SOC was retrieved from the chat history stored in the intervention system. The number of intervention sessions completed by all participants was also documented.

### Statistical Analysis

There were no missing data for participants who had completed the surveys at baseline and month 6. The proportion of missing data at month 6 was equal to the dropout rate. Both complete case and intention-to-treat (ITT) analyses were performed. In the ITT analysis, missing data on the primary outcome (SIV uptake) were treated as *does not happen* to allow a conservative estimation of the efficacy of the intervention. Assuming that the data were missing at random, a Markov chain Monte Carlo method was used to impute missing values of the SOC at month 6 separately by randomized group.^21^ Variables used to impute the missing values included baseline values of the SOC. The χ^2^ test or independent-sample unpaired, 2-tailed *t* tests were used to inspect between-group balances of baseline characteristics. The relative risk (RR), absolute risk reduction (ARR), and number needed to treat (NNT) and their respective 95% CI were calculated using Excel. Among participants in the intervention group who completed at least 2 sessions, differences between their first SOC and last SOC were compared using pair-sample *t* tests. Two-sided *P* < .05 indicated statistical significance. Statistical analysis was performed with SPSS, version 26.0 (IBM SPSS).

## Results

**Figure 3.**
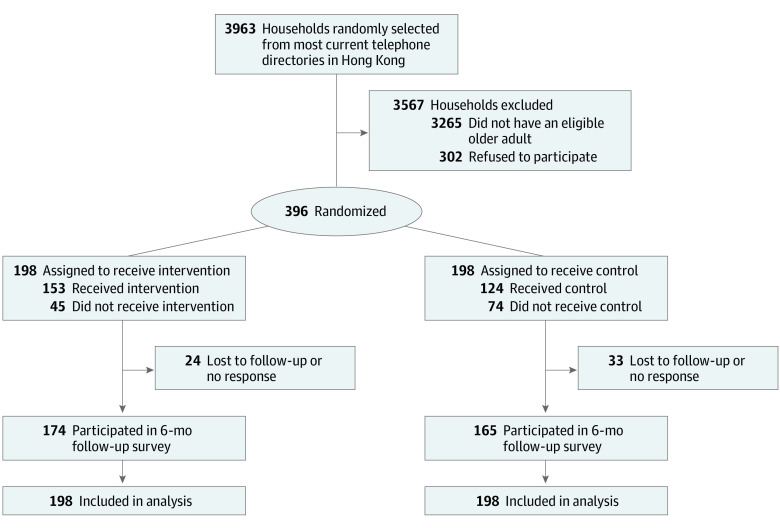
Study Flow Diagram

In total, 3963 households were contacted, 698 of which had eligible participants; 302 individuals refused to participate in the trial due to time or other logistical reasons, and 396 completed the baseline survey (Figure 3). Participants who completed the survey were randomized to either the intervention (n = 198) or control (n = 198) group and included 249 females (62.9%) and 147 males (37.1%) with a mean (SD) age of 70.2 (4.3) years. At baseline, 237 participants (59.8%) had ever received a SIV. Regarding SOC, 148 participants (37.4%) were at precontemplation stage, 87 (22.0%) were at contemplation stage, and 161 (40.6%) were at preparation stage. No significant between-group difference in baseline characteristics was found (Table 1). The dropout rate was 14.4% at month 6. Comparison of baseline characteristics of dropouts and nondropouts is provided in eAppendix 3 in Supplement 2.

**Table 1.** Characteristics of Participants at Baseline

Characteristic	Participants, No. (%)		
	All	Intervention group	Control group
All participants	396 (100)	198 (50.0)	198 (50.0)
Sociodemographic characteristics			
Age group, y			
65-69	201 (50.8)	104 (52.5)	97 (49.0)
70-74	134 (33.8)	65 (32.8)	69 (34.8)
≥75	61 (15.4)	29 (14.7)	32 (16.2)
Sex			
Male	147 (37.1)	81 (40.9)	66 (33.3)
Female	249 (62.9)	117 (59.1)	132 (66.7)
Relationship status			
Single	106 (26.8)	47 (23.7)	59 (29.8)
Married or cohabiting with a partner	290 (73.2)	151 (76.3)	139 (70.2)
Educational level			
≤Primary	164 (41.4)	86 (43.4)	78 (39.4)
Secondary	189 (47.7)	89 (44.9)	100 (50.5)
≥Tertiary	43 (10.9)	23 (11.7)	20 (10.1)
Monthly household income, HK$ (US $)			
<20 000 (2580)	294 (74.2)	144 (72.8)	150 (75.8)
≥20 000 (2580)	52 (13.2)	27 (13.6)	25 (12.6)
Refuse to disclose	50 (12.6)	27 (13.6)	23 (11.6)
Receiving CSSA			
No	366 (92.4)	185 (93.4)	181 (91.4)
Yes	30 (7.6)	13 (6.6)	17 (8.6)
Living alone			
No	321 (81.1)	158 (79.8)	163 (82.3)
Yes	75 (18.9)	40 (20.2)	35 (17.7)
Lifestyle and health conditions			
Smoking in past year			
No	369 (93.2)	185 (93.4)	184 (92.9)
Yes	27 (6.8)	13 (6.6)	14 (7.1)
Binge drinking in past year			
No	387 (97.7)	194 (98.0)	193 (97.5)
Yes	9 (2.3)	4 (2.0)	5 (2.5)
Presence of chronic condition: yes			
Hypertension	189 (47.7)	100 (50.5)	89 (44.9)
Chronic CVD	42 (10.6)	19 (9.6)	23 (11.6)
Chronic lung diseases	8 (2.0)	6 (3.0)	2 (1.0)
Chronic liver diseases	8 (2.0)	5 (2.5)	3 (1.5)
CKD	3 (0.8)	2 (1.0)	1 (0.5)
Diabetes	75 (18.9)	39 (19.7)	36 (18.2)
Any of the above	239 (60.4)	127 (64.1)	112 (56.6)
History of COVID-19			
No	390 (98.5)	196 (99.0)	194 (98.0)
Yes	6 (1.5)	2 (1.0)	4 (2.0)
Vaccination history			
History of SIV			
No	159 (40.2)	73 (36.9)	86 (43.4)
Yes	237 (59.8)	125 (63.1)	112 (56.6)
No. of SIV doses in past 3 y			
0	180 (45.5)	86 (43.4)	94 (47.5)
1	33 (8.3)	14 (7.1)	19 (9.6)
2	48 (12.1)	21 (10.6)	27 (13.6)
3	135 (34.1)	77 (38.9)	58 (29.3)
History of pneumococcal vaccination			
No	293 (74.0)	145 (73.2)	148 (74.7)
Yes	103 (26.0)	53 (26.8)	50 (25.3)
No. of doses of COVID-19 vaccination			
0	153 (38.7)	76 (38.4)	77 (38.9)
1	8 (2.0)	3 (1.5)	5 (2.5)
2	235 (59.3)	119 (60.1)	116 (58.6)
SOC			
SOC related to SIV^a^			
Precontemplation stage	148 (37.4)	64 (324)	84 (42.4)
Contemplation stage	87 (22.0)	48 (24.2)	39 (19.7)
Preparation stage	161 (40.6)	86 (43.4)	75 (37.9)
SOC score, mean (SD)^b^	2.0 (0.9)	2.1 (0.9)	2.0 (0.9)

Abbreviations: CKD, chronic kidney disease; CSSA, Comprehensive Social Security Assistance; CVD, cardiovascular disease; SIV, seasonal influenza vaccination; SOC, stage of change.

^a^ Precontemplation stage was defined as without intention to obtain SIV in the next 6 months; contemplation stage was defined as intention to obtain SIV in the next 6 months but without a plan to do so in the next month; and preparation stage was defined as intention to obtain SIV within the next month. Those who had already received SIV for the incoming influenza season were at action stage.

^b^ 1 = Precontemplation stage, 2 = contemplation stage, 3 = preparation stage, and 4 = action stage. Independent-sample, unpaired, 2-tailed *t* test was used to compare the SOC scores between the intervention group and the control group.

At month 6, 100 participants (50.5%) in the intervention group and 70 participants (35.3%) in the control group reported receiving a SIV during the study period, and all of them provided SIV receipts for verification. The SIV uptake rate in the intervention group was significantly higher than that in the control group (complete case analysis: 57.5% vs 42.4%, RR = 1.35 [95% CI, 1.09-1.69], ARR = 0.15 [95% CI, 0.05-0.26], NNT = 6.6 [95% CI, 3.9-22.1], *P* = .006; ITT analysis: 50.5% vs 35.3%, RR = 1.43 [95% CI, 1.13-1.80], ARR = 0.15 [95% CI, 0.06-0.25], NNT = 6.6 [95% CI, 4.0-18.1], *P* = .002). The mean (SD) SOC score was higher in the intervention group than in the control group (complete case analysis: 2.8 [1.4] vs 2.4 [1.4], *P* = .01; ITT analysis: 2.8 [1.4] vs 2.4 [1.4], *P* = .02) (Table 2). Among 117 participants in the intervention group who completed at least 2 intervention sessions, 65 (55.6%) progressed to a higher SOC when comparing their data measured at the last vs the first intervention session. The increase in mean (SD) SOC score was statistically significant (2.8 [1.3] vs 2.2 [0.9]; *P* < .001) (eAppendix 4 in Supplement 2).

**Table 2.** Between-Group Difference in SIV Uptake and SOC at Month 6

Outcome	Participants, No. (%)		*P* value
	Intervention group	Control group	
**Complete case analysis**			
Total participants	174	165	NA
SIV uptake: yes	100 (57.5)	70 (42.4)	.006
SOC at month 6			
Precontemplation stage	58 (33.3)	73 (44.2)	NA
Contemplation stage	16 (9.2)	17 (10.3)	NA
Preparation stage	0	5 (3.0)	NA
Action stage	100 (57.5)	70 (42.4)	.008
SOC score, mean (SD)^a^	2.8 (1.4)	2.4 (1.4)	.01
Changes in SOC score from baseline to month 6, mean (SD)^a^	0.7 (1.0)	0.4 (1.0)	.048
**Intention-to-treat analysis**			
Total participants	198	198	NA
SIV uptake: yes	100 (50.5)	70 (35.3)	.002
SOC at month 6			
Precontemplation stage	68 (34.3)	88 (44.4)	NA
Contemplation stage	18 (9.1)	21 (10.6)	NA
Preparation stage	0 (0)	9 (4.5)	NA
Action stage	112 (56.6)	80 (40.4)	.001
SOC score, mean (SD)^a^	2.8 (1.4)	2.4 (1.4)	.02
Changes in SOC score from baseline to month 6, mean (SD)^a^	0.7 (1.0)	0.5 (1.0)	.04

Abbreviations: NA, not applicable; SIV, seasonal influenza vaccination; SOC, stage of change.

^a^ 1 = Precontemplation stage, 2 = contemplation stage, 3 = preparation stage, and 4 = action stage. The χ^2^ tests were used to compare SIV uptake and SOC between the intervention group and the control group. Independent-sample, unpaired, 2-tailed *t* test was used to compare the SOC score and changes in the SOC score between the intervention group and the control group.

As documented by the system, more participants in the intervention group completed at least 1 episode of intervention than that of the control group (77.3% vs 62.6%; *P* < .001). The mean (SD) number of intervention sessions completed by the participants was 1.6 (1.4) in the intervention group and 1.6 (1.6) in the control group (eAppendix 5 in Supplement 2). Approximately 80% of participants in the intervention group found it easy to interact with the intervention system and were satisfied with the health promotion messages (eAppendix 6 in Supplement 2).

## Discussion

This trial contributed to the literature by evaluating the efficacy of a theory-based online intervention and developing new methods to increase SIV uptake among adults 65 years or older. In line with our hypothesis, the SOC-tailored intervention was more effective than the non–SOC-tailored intervention in increasing SIV uptake among participants. The SIV uptake rate in the intervention group was 10 percentage points higher than the overall SIV coverage among all Hong Kong residents 65 years or older in the same influenza season (50.5% vs 40.4%).^8^ The process evaluation indicated that a messaging application–based intervention system was viable in providing health promotion messages that were tailored to the needs of adults 65 years or older at different SOC. Since the intervention system could automatically assess users’ SOC and select a pathway for interventions, it required fewer resources to implement.

Several reasons might explain the higher SIV uptake in the intervention group. First, as documented by the intervention system, the SOC-tailored intervention helped in facilitating the intervention group to progress to a higher SOC, which might lead to the observed behavioral changes. Second, online interventions for the intervention group were guided by behavioral change strategies recommended by the TTM, whereas those for the control group did not involve such strategies. Theory-based interventions were more effective than non–theory-based interventions in facilitating behavioral changes.^17^ Third, the duration of the online videos in the intervention group was slightly longer than that in the control group. Videos for the intervention group contained more information. Moreover, the participants had higher compliance to the SOC-tailored intervention than non–SOC-tailored intervention. The intervention system proactively interacted with participants in the intervention group, which could improve compliance.^22^ In addition, interventions that were tailored to users’ SOC were more likely to create personal relevance, an essential component for intrapersonal health communication.^23^

### Limitations

This study has several limitations. First, we did not have a control group without using the intervention system. This trial could not provide evidence to support whether the intervention delivered by the simplified rule-based chatbot integrated with the messaging application was better than the intervention without a chatbot. The simplified rule-based chatbot might not meet the most updated definition of a chatbot.^11^ Second, the COVID-19 pandemic and COVID-19 vaccination rollout might have affected the study outcome. The pandemic did not affect participant recruitment and intervention delivery. No suspension or interruption of SIV services was reported during the study period. However, it was possible that some adults 65 years or older missed their SIV appointment due to COVID-19 infection. Since the Hong Kong government suggested a 14-day interval between COVID-19 vaccination and SIV,^24^ some of the target population might have had to reschedule their SIV appointment due to timing. The effects of COVID-19 vaccination were expected to be similar between the 2 study groups, as we used RCT as the evaluation design. Third, the intervention was limited to adults 65 years or older who had access to smartphones. In Hong Kong, smartphone ownership among adults in this age group has been high (73% in 2021) and increasing sharply.^25^ Fourth, people who were 75 years or older were undersampled in this study.^26^ Fifth, the trial was conducted in Hong Kong, and findings may not be directly applicable to other populations. Sixth, participants and those who refused to participate might have different motivations to receive a SIV and different characteristics, and self-selection bias existed. Seventh, lack of blinding might lead to overestimation of the intervention effects. Moreover, the dropouts in the intervention group were less likely to report a history of SIV at baseline than nondropouts. The SIV uptake might be overestimated in the intervention group. Furthermore, the follow-up period was relatively short, limiting our observation of the long-term effects of the interventions. Eighth, SOC was mainly self-reported and could not be validated.

## Conclusions

In this RCT, SOC-tailored online intervention was more effective than the non–SOC-tailored intervention in increasing the SIV uptake among Hong Kong residents 65 years or older. Interventions tailored to SOC might facilitate participants to progress to a higher SOC. Adults 65 years or older also had higher compliance to the SOC-tailored intervention. These findings suggest that SOC-tailored interventions may be a sustainable new method for increasing SIV uptake for this population.

## Notes

Supplement 1.
Trial Protocol

Supplement 2. **eAppendix 1.** Development of Intervention Materials **eAppendix 2.** Development and Maintenance of the Intervention System **eAppendix 3.** Comparing Baseline Characteristics Between Participants Who Completed Month 6 Evaluation and Those Who Were Lost to Follow-Up **eAppendix 4.** Changes in Stage of Changes Documented by the Chatbot Among Participants Who Had Completed at Least Two Intervention Sessions in the Intervention Group **eAppendix 5.** Compliance to the Interventions **eAppendix 6.** Process Evaluation

Supplement 3.
Data Sharing Statement
