# Supplementary material for: Chatbot-Delivered Online Intervention to Promote Seasonal Influenza Vaccination During the COVID-19 Pandemic: A Randomized Clinical Trial
Source: JAMA Netw Open. 2023 Sep 11;6(9):e2332568. doi: 10.1001/jamanetworkopen.2023.32568 (PMC10495860; doi:10.1001/jamanetworkopen.2023.32568)
Supplement: Supplement 1. — Trial Protocol [file jamanetwopen-e2332568-s001.pdf]

Section 13 PROPOSED RESEARCH PROJECT

This template applies to the following Area of Project –

- **Public health, human health and health services research**
- **Infectious diseases**
- **Advanced medical research**

a) Title:
A randomized controlled trial evaluating an online intervention based on the Trans-Theoretical Model in increasing seasonal influenza vaccination among community dwelling people aged ≥65 years

b) Introduction:
Seasonal influenza --- a serious public health threat

Seasonal influenza causes symptoms such as sudden onset of fever, cough, headache, muscle and joint pain, severe malaise, sore throat and running nose (1). Although most people may recover from these symptoms without requiring medical attention, it can cause severe illness or death, especially among elderly and children (1). Worldwide, annual seasonal influenza epidemics cause 3-5 million cases of severe illness, and 290,000-650,000 deaths (1). In Hong Kong, the flu season usually lasts from January to March and from July to August (2). Severe cases and deaths caused by seasonal influenza mainly affected individuals aged ≥65 years. During the 2017/18 winter flu season, individuals aged ≥65 years accounted for about 72% of severe cases and 87% of deaths caused by seasonal influenza (3). This group also had the second highest weekly admission rate with principal diagnosis of influenza in public hospitals (4.25/10,000) during the 2017/18 winter flu season (3).

Seasonal influenza vaccination (SIV) for elderly --- an important health initiate

When the vaccine strains closely matched with the circulating influenza virus, SIV could offer 49% protection in preventing influenza and 44-61% protection in preventing all-cause mortality among individuals aged ≥65 years (4, 5). Taking up SIV prior to the flu season offers better protection as the body takes about two weeks to develop antibodies after vaccination (2). Getting SIV later can still be beneficial (6). Taking up SIV once is expected to gain protection for both winter and summer flu seasons (2). Consistently, cost-effectiveness for free SIV for community dwelling elderly aged ≥65 years has been established (7).

International and local SIV policy and programs for elderly

The World Health Organization (WHO) strongly recommends individuals aged ≥65 years to receive annual SIV due to their high risk of complications and excess hospital admission and deaths from seasonal influenza. The WHO concludes that SIV is the most efficacious public health tool currently to prevent elderly against influenza. In line with the WHO, the Hong Kong Centre for Health Protection (CHP) also recommends all individuals aged ≥65 years to receive SIV once every year (2).

Many countries offer free SIV to elderly (e.g., U.S., mainland China, etc.). The Hong Kong Government Vaccination Program (GVP) are offering free SIV to all local residents aged ≥65 years at

public general outpatient clinics and designated elderly health care centres (8). Elderly residents aged ≥65 years can also receive subsidized SIV from private doctors under the Vaccination Subsidy Scheme (VSS). The subsidy was HK$210 per dose for SIV in 2018. The GVP and VSS for SIV usually launch in October. The CHP encourages individuals to receive SIV at least two weeks before the winter flu season to maximize the protective effect, however, SIV is offered throughout the flu season (2).

Inadequate coverage of SIV among individuals aged ≥65 years in Hong Kong

It was estimated that the SIV coverage among individuals aged ≥65 years was 40.8% in 2016-2017 (9). Such coverage was lower than other developed regions/countries (49.2% in Taiwan, 70.5% in England and 65.3% in the U.S.) (9). Since SIV is routinely offered to elderly residents of residential care homes and elderly in-patients in public hospitals, the coverage of SIV may be lower among community dwelling elderly (2). There is urgent need and much room for improvement.

Factors associated with SIV uptake among community dwelling elderly

Factors influencing SIV uptake among elderly are well studied. A recent published systematic review on this topic included 36 studies, seven of them were conducted in Hong Kong (10). Some factors have consistently been documented as determinants of SIV uptake: 1) socio-demographics (e.g., age, gender, whether living with family members), 2) health status (e.g., presence of chronic diseases, physical function, and history of influenza), 3) lifestyle and medical service utilization (e.g., smoking, recent visit of clinics, history of other vaccination), 4) knowledge related to SIV, and 5) perceptions related to SIV (e.g., perceived risk of contracting influenza, belief that influenza would cause severe complications or have impact on daily activities, perceived efficacy of SIV, concerns about potential side-effects of SIV, perceived transportation inconvenience/long traveling time for taking up SIV, and belief that doctors/nurses and other significant others would support them to get vaccinated).

Interventions promoting SIV among community dwelling elderly

There are a number of community-based randomized controlled trials (RCT) promoting SIV among community dwelling elderly, while several others were targeting elderly patients in clinic settings (11). These RCTs yielded mixed results in increasing SIV uptake. There were a few RCTs comparing mailing of simple reminder letters/postcards/leaflets versus no intervention, only half of them were effective. These significant RCTs reported small effective size (4.8-9.8%) (11). More intensive interventions (e.g., telephone education session delivered by nurses or home visit paid by nurses/volunteers) were more likely to be effective and had larger effect size (11). However, such interventions required a large number of phone/face-to-face education sessions by nurses/volunteers, making them less sustainable. In Hong Kong, we could only find one RCT promoting SIV targeting elderly outpatients instead of community dwelling elderly. Those in the intervention group received one-to-one face-to-face verbal education by medical students, and the control group received no intervention. The intervention yielded a SIV uptake rate of 33.6% (control: 25%, $p<0.05$) but required considerable manpower to implement. It was hence less sustainable (12).

Although theory-based interventions are more effective than non-theory-based ones (13), none of the aforementioned intervention was based on behavioural health theories. Furthermore, online intervention has the advantages of reaching out to general public at low cost and allowing users to access information easier and quicker, it has not been used to promote SIV among elderly.

Online intervention is feasible and effective for health promotion among elderly

In literature, online interventions were shown to be feasible and effective in promoting physical activities and mental health, and improving hypertension self-care among elderly [e.g., (14)]. Official data showed that smartphone ownership among residents aged ≥60 years was about 65% in 2017 and has been increasing sharply over time (increased by about 10% per year). In our pilot study (n=50), 70% of elderly aged ≥65 years had smartphones. It is hence feasible to develop online intervention promoting SIV among local elderly.

Stage-tailored and theory-based interventions promoting SIV among elderly

The Trans-Theoretical Model (TTM) has been extensively used to guide behavioural change for health promotion (15). It conceptualizes the process of behavioural change. Stage of change (SOC) is the core of TTM, is a measure of readiness for behavioural change (Figure 1) (15). SOC postulates that completed behavioural changes need to go through five ordinal stages: 1) pre-contemplation stage (do not intend to take action in the foreseeable future), 2) contemplation stage (intend to change in the foreseeable future), 3) preparation stage (intend to take action in the immediate future), 4) action stage, and 5) maintenance stage.

Meta-analysis showed that interventions tailored to one's current SOC are more effective than non-stage-tailored, especially among less-motivated individuals (16). Stage-tailored interventions based on the TTM had been successfully used to promote screening behaviours, smoking cessation, physical activities, dietary change, condom use and substance use, some of these interventions were conducted among elderly (17). To our knowledge, only one study applied stage-tailored interventions to promote vaccination (HPV vaccination) (18).

In addition, studies showed people might move forward the later SOC, go backward earlier SOC, or stay in the same SOC after exposing to health promotion. Therefore, it is highly recommended that stage-tailored interventions should have multiple sessions and each session should tailor to people's current SOC (19). Very few stage-tailored interventions based on the TTM had more than one session.

Strategies facilitating transition of SOC

Studies showed that changes in SOC mediated the TTM intervention effects (20). SIV uptake is different from many other health behaviours with regard to SOC because it is one-off, making action and maintenance stage less meaningful. This study will focus on pre-contemplation, contemplation and preparation stage. According to TTM, different strategies are recommended for people in different SOC (15):

1) **To facilitate transition from pre-contemplation stage to contemplation stage,** common

strategies include increase their awareness of potential behavioural changes by providing information and explaining reasons for making changes.

2) **To facilitate transition from contemplation stage to preparation stage,** strategies include elicit pros and cons to shift their decisional balance in favour or pros, make specific suggestion, and encourage to make specific plan for taking action.

3) **To facilitate transition from preparation stage to action stage,** strategies include assist them develop and implement concrete action plan in order to increase their self-efficacy.

Fully-automated conversational agent as a cost-effective means to deliver stage-tailored interventions promoting SIV for elderly

Computer-aided systems are potential cost-effective means to deliver interventions promoting SIV (21). A conversational agent (the "Chatbot") is a software program that can automatically select and provide different path of interventions according to participants' responses. A review suggested that conversational agents were feasible and effective in delivering interventions promoting physical activities, healthy diets, and smoking cessation (22).

In collaboration with IT engineers (Co-I: Prof. QP Zhang), our team has developed a special "Chatbot" that was applied in smoking cessation (23). It is innovative for integrating a conversational agent with an instant messaging platform (WeChat). The "Chatbot" can assess users' current smoking status through WeChat, and then automatically disseminate WeChat interventions (either text messages, images or videos) tailored to current status. For example, the "Chatbot" asked participants to clinic a box indicating number of cigarette consumed in the past week. For those click zero consumption, the "Chatbot" will automatically send health promotion messages strengthening maintenance of quitting. While for those with cigarette smoking, the "Chatbot" will send messages to motivate them to quit. The details of this system was described by a recently published paper (23).

The "Chatbot" is potentially useful to deliver stage-tailored online intervention promoting SIV among elderly. With some modification, it can assess users' SOC regarding SIV uptake and disseminate tailored interventions through instant messaging platforms. Since it is fully-automated and has virtually no maintenance cost, it is especially suitable and cost-effective to deliver multiple sessions of stage-tailored intervention. To our knowledge, this approach is novel.

Significance

Although efforts of promoting SIV among community dwelling elderly existed, they were either less effective or too resource-demanding to sustain. None of them was based on behavioural health theories or made use of online intervention. This study is novel for applying TTM-based intervention tailored to elderly individuals' SOC regarding SIV uptake, which will increase its efficacy. Making use of a fully-automated "Chatbot" to deliver stage-tailored intervention is also innovative. Such approach can reach out to general public at low cost and is potential sustainable.

c) Aims and Hypotheses to be Tested:

The proposed RCT will evaluate the relative efficacy of a stage-tailored online intervention based on

the TTM versus a control in promoting uptake of SIV among people aged ≥65 years. The stage-tailored online intervention (ST group) is delivered by a fully-automated "Chatbot". According to participants' answers to simple questions assessing their SOC, the "Chatbot" will automatically select and send them one of the online health promotion videos tailored to their SOC through WhatsApp once every two weeks for four times. The control group will watch a short online video covering general information about SIV every two weeks for four times.

We hypothesize that interventions in the ST group will result in higher uptake of SIV during the study period as compared to the control group.

d) Plan of Investigation:

(i) Subjects

Inclusion criteria are: 1) aged ≥65 years, 2) having Hong Kong ID, 3) Chinese speaking, 4) willing to be followed up by telephone, 5) having a smartphone, and 6) have not received SIV for the incoming flu season.

Exclusion criteria include: 1) cognitive impairment, blindness or deafness, 2) not able to communicate with others effectively, and 3) with known contradictions of SIV listed by the CHP (allergic to previous SIV, diagnosed/suspected egg allergy, with bleeding disorder or on warfarin)

(ii) Methods

Recruitment procedures

Participants will be recruited through random telephone sampling; **the method was used for recruitment local elderly in our intervention study (HMRF 15161231)**. Telephone numbers will be selected from up-to-date Hong Kong telephone directories. Trained interviewers will conduct the telephone calls. If there is more than one person in the household who is aged ≥65 years, the one whose last birthday is closest to the interview date will be invited to join the study. This is to avoid contamination and introduction of extra confounding factors. Eligibility will be screened.

Prospective eligible participants will be briefed about the study. Guarantees will be made on anonymity, right to quit at any time and that refusal will not affect their chance in using services. Since there will not be face-to-face contact and the study is anonymous, the interviewers will sign a form pledging that the participants have been fully informed about the study. The procedure has been used in studies involving online interventions without face-to-face contact (HMRF 13141651 & 15161231). Ethics approval will be obtained from the Survey and Behavioral Research Ethics Committee of the Chinese University of Hong Kong and the joint Chinese University of Hong Kong --- New Territories East Cluster Clinical Research Ethics Committee.

In our pilot study, we have successfully recruited 50 elderly individuals through random

telephone sampling; 80% were willing to receive interventions promoting SIV via smartphones (receive links to watch online videos at baseline, and 2, 4 and 6 weeks afterwards). Recruitment took four weekdays and one weekend. It is hence feasible to recruit 396 participants within 4 months.

Randomization and baseline survey

Participants will be interviewed through telephone to record background characteristics at baseline. At the end of baseline survey, the interviewers will help participants install WhatsApp if needed, have their WhatsApp connected to the Chatbot, and provide instruction on how to use WhatsApp and Chatbot. Participants will be randomized evenly either to the ST group or the control group by a randomization algorithm built into the Chatbot. The automated randomization occurs online and is concealed from the research team.

Intervention for the ST group

Overview

Participants will be exposed to one of the online health promotion videos tailored to their SOC regarding SIV uptake once every two weeks for four sessions through WhatsApp. The "Chatbot" that was applied in smoking cessation will be modified to disseminate stage-tailored interventions for this study (23).

Set up the intervention at baseline

At the end of the baseline survey, participants will be briefed by the interviewers that an automated computer program will ask them some simple questions related to SIV uptake and send them a link to access an online video once every two weeks.

Identification of SOC and initiation of intervention at week 0, 2, 4 and 6

At the beginning of each session, the "Chatbot" will assess participants' SOC by asking participants to fill out a simple online questionnaire (i.e., whether he/she intends to take up SIV in the next six months, and whether he/she plans to do so in the next month). Pre-contemplation stage is defined as not intending to take up SIV in the next six months, contemplation stage is defined as intending to take up SIV in the next six months but without plans to do so in the next month, while preparation stage is defined as having plans to take up SIV in the next month.

Starting from Week 2, an additional question will be asked to confirm whether the participant has already taken up SIV for the incoming flu season. For those who click "yes", the "Chatbot" will make a record and terminate the program automatically. For those who have not yet taken up SIV, the "Chatbot" will automatically sent them a link to access one of the health promotion videos corresponding to their SOC through WhatsApp.

Contents of the online videos tailored to each SOC

1) Pre-contemplation stage: the video aims to increase participants' awareness about the importance of taking up SIV. The video will be presented by a primary care physician (Prof.

Martin Wong, our Co-I) covering the following topics: a) information about high risk of influenza infection and severe consequences among elderly, b) SIV is an effective means to protect elderly from seasonal influenza, and c) free SIV vaccination is available for elderly.

2) Contemplation stage: the video aims will cover the following contents.

(a) Increase perceived pros: The same physician will talk about promising efficacies of SIV in preventing seasonal influenza and reducing related hospitalization and deaths, and protect family members through herd immunization. Elderly who have taken up SIV will share about positive experience related to SIV (the feeling of relief/secure after receiving SIV).

(b) Reduce perceived cons: The same physician will explain common side-effects of SIV are mild and severe side-effects are rare. Testimonials of vaccinated elderly regarding side-effects will also be presented.

(c) Provide suggestion and encourage to make specific plan: The same physician will give recommendation of and encourage elderly to make plan to receive SIV before mid-December. Expert recommendation was a strong facilitator of SIV uptake (24)

3) Preparation stage: the video aims to assist participants to develop and implement concrete action plan to increase their perceived self-efficacy related to SIV. The video will cover: a) location and contact of facilities offering free SIV for elderly in different districts, and b) contacts and procedures of making appointment. At the end, participants will be asked to click a box to indicate when and where they decide to take up SIV. Reminder messages will be automatically sent to them by the “Chatbot” twice a week to reinforce their self-efficacy.

In order to avoid participant watching a same video twice. Four slightly different online videos corresponding to each SOC will be prepared. In each session, the “Chatbot” will randomly select one for participants.

Intervention for the control group

The “Chatbot” will automatically send a link to access a short online video (about 2 minutes) covering basic information about SIV (who, when and where to receive SIV) at week 0, 2, 4 and 6.

During the intervention period, both groups can contact our project staff through the hotline.

Development of intervention materials

A panel consisting of the investigators (public health researchers, experts in behavioural health, health psychologist, and family medicine physician) and two local elderly people will be formed. Discussion groups of local elderly will be conducted to inform the design of interventions.

Fidelity checks

To ensure exposure, all videos will be formatted in a way that participants cannot fast-forward.

The system will automatically record time between starting and leaving the four video-links. We have used such checks in previous projects (HMRF 13141651).

Measures to avoid contamination

First, the access to the online video will be valid for a relatively short period of time and can only be used once (request is required for re-entry). The videos cannot be downloaded by the participants. Second, only one member per household will be recruited, the within-household contamination is hence avoided. Third, since the recruitment is based on population-based random telephone contacts, the chance that two participants knowing each other and sharing the intervention materials is extremely rare and not of practical concern.

Measures to reduce drop-out rate

First, participants are invited to leave multiple contacts (mobile, social media, email, etc.) during the recruitment. Second, interviewer will obtain participants' preferred date and timeslot for follow-up surveys after completion of the baseline survey. Reminders will be sent to them one month, two weeks, one week, and one day prior to the scheduled date of follow-up surveys by SMS/social media/email. If necessary, we will extend the recruitment period. Our previous RCT intervention studies targeting elderly individuals had very low drop-out rate (HMRF 14153321: 13.9% at Month 3). Therefore, we believe our assumption of 30% drop-out rate at Month 6 should be conservative.

Measurements

Primary outcome: prevalence of self-reported uptake of one dose of SIV within 6 months after intervention is the primary outcome. This outcome will be validated by requesting participants to upload an image of the receipt after taking up SIV, hiding personal identification and via the same WhatsApp account used in this project. No incentive will be offered for validating SIV uptake. Weekly reminders will be sent to them if they fail to do so. Same verification procedure for vaccination uptake has been used in our HMRF project (13141651).

Secondary outcome: SOC related to SIV measured at baseline and 6 months after intervention. Pre-contemplation stage is defined as not intending to take up SIV in the next six months, contemplation stage is defined as intending to take up SIV in the next six months but without plans to do so in the next month, while preparation stage is defined as having plans to take up SIV in the next month. Those who have taken up SIV without the follow-up period are at the action stage.

Background variables and potential confounders: socio-demographics, presence of chronic conditions, history of SIV and knowledge related to SIV will be measured at baseline survey.

Incentives for the surveys

A supermarket coupon (HK$50) will be mailed to an address provided by the participant upon completing the baseline and Month 6 follow-up surveys. The purposes of offering such

incentives are to ensure response rate and as an appreciation for their time spent (participants have to spend time making appointment and about 20 minutes to complete each survey). Same amount of incentive for completing baseline/follow-up surveys was used in RCT targeting elderly in Hong Kong (HMRF 14153321 & 15161231).

Pilot study

A pilot study of six eligible participants randomized into the two groups will be conducted to test the logistics of the intervention. Refinements will be made if necessary.

Sample size planning

Our pilot data showed that 50% of the participants intended to complete take up SIV in the next six months after being briefed about some facts of SIV. The prevalence of behavioural intention is similar to other studies conducted in Hong Kong (24). Meta-analysis showed that 43-62% of those with a behavioural intention would translate it into action. For planning purposes, we conservatively assume 50% of those in the control group would show an intention, and 40% of those with such an intention would take up SIV (20% of the control group). For planning purpose, we use smallest detectable difference of 15% between the ST group and the control group (35% in the ST group). We need 138 per group to achieve planned effect sizes and power of 0.8 and alpha of 0.05. Assume that the loss-to-follow-up rate will be 30% at Month 6, a total of 198 participants per group will be required. The total sample size will be 396 (PASS 11.0; NCSS; Kaysville; U.S.).

(iii) Study design A non-blinded 2-arm parallel RCT will be conducted (See flowchart in Figure 2). Participants will be interviewed by phone at baseline, and 6 months after completion of intervention. Participants present at Month 6 will be asked a final evaluation questionnaire recording uptake of SIV during the follow-up period and the secondary outcome.

(iv) Data processing and analysis Intention-to-treat analysis (ITT) will be performed. Missing data on the primary outcome (SIV uptake) will be treated as “does not happen”, a standard method in ITT analysis, in order to provide a conservative estimation of the efficacy of the intervention (25). Assuming the data are missing at random, a Markov Chain Monte Carlo Method will be used to impute missing continuous secondary outcome (SOC). Between-group comparisons will be made. Chi-square test or independent-sample t tests will be used to inspect between-group balances of potential confounders at baseline, and adjustment will be made if any potential confounders show $p<.05$ in the comparisons. The relative risk reduction, absolute risk reduction, and number need to treat and their 95% confidence interval will be reported.

[Word Count: 4000]

e) Impact on People’s Health and Health Services as well as Plan to Disseminate Research Findings to End Users:

The underlying purpose of the study is to prevent seasonal influenza and its complications by promoting uptake of seasonal influenza vaccination (SIV) among community dwelling elderly aged ≥65 years. In Hong Kong, seasonal influenza is a serious public health threat for elderly causing large number of severe cases/deaths and hospital admissions every year. Although SIV is recommended and provided for free to eligible older people in Hong Kong, the coverage of SIV in this group was relatively low (40% among all people aged ≥65 years). International experiences showed that SIV

uptake among elderly in countries where SIV is free and recommended but without effective interventions may only be moderate.

The proposed intervention has strong applications to infectious diseases control in Hong Kong. SIV is the most efficacious public health tool to prevent elderly from seasonal influenza. Although efforts promoting SIV among elderly existed both locally and internationally, effective and sustainable interventions are greatly needed but lacked. We will develop an online intervention based on the Trans-theoretical model and compare its efficacy with the control group by using randomized controlled trial, which is the strictest evaluation method giving evidence of the highest quality. Our intervention is tailored to participants' current stage of change (SOC) regarding SIV uptake. Stage-tailored interventions are more effective. Considering participants may move forward to a later SOC, go backward to an earlier SOC, or stay in the same SOC after exposing to an intervention. Our intervention has multiple sessions, and each session will tailor to their current SOC. This approach will increase the efficacy. All intervention will be delivered by a fully-automated "Chatbot" which integrates a conversation agent with WhatsApp. It can identify respondents' SOC related to SIV uptake and automatically deliver intervention corresponding to their current SOC. Since the "Chatbot" has virtually no maintenance cost, it is especially suitable and cost-effective to deliver our proposed intervention. The sustainability of our intervention is expected to be high. The intervention, if found to be significant and translated into service, would increase coverage of SIV among elderly and enhance effectiveness of the governmental SIV programs.

Besides practical significance of improving SIV coverage, the study is novel in applying Trans-Theoretical Model and making use of artificial intelligence. There were only a few community-based RCT conducted for this important subject in Chinese population. This study also adds the literature of interventions on SIV promotion.

Reports will be sent to the Department of Health, Hospital Authority for reference, university departments, and non-governmental organizations (NGOs) providing elderly health services. We will organize seminars to discuss about the findings with researchers, nurses, physicians and NGO workers working in related areas. The findings will also be presented in local and international meetings and published in international journals.

f) Key References:

1. World Health Organization. Fact Sheets: Influenza (seasonal). Available at: https://www.who.int/news-room/fact-sheets/detail/influenza-(seasonal). 2018.

2. Centre for Health Protection. Recommendation on Seasonal Influenza Vaccination for the 2018/19 season in Hong Kong. Available at: https://www.chp.gov.hk/files/pdf/scvpd_recommendations_on_siv_for_2018_19_season.pdf. 2018.

3. Centre for Health Protection. Seasonal influenza vaccination & pneumococcal vaccination. Available at: https://www.chp.gov.hk/files/pdf/1sivandpv.pdf. 2018.

4. Lang PO, Mendes A, Socquet J, Assir N, Govind S, Aspinall R. Effectiveness of influenza vaccine in aging and older adults: comprehensive analysis of the evidence. Clinical interventions in aging. 2012;7:55-64.

5. Wilkinson K, Wei Y, Szwajcer A, Rabbani R, Zarychanski R, Abou-Setta AM, et al. Efficacy and safety of high-dose influenza vaccine in elderly adults: A systematic review and meta-analysis. Vaccine. 2017;35(21):2775-80.

6. Center for Disease Control and Prevention. Misconceptions about flu vaccines. Available at: https://www.cdc.gov/flu/about/qa/misconceptions.htm. 2018.

7. Schooling CM, Wong LC, Chau J, Cheung A, Ho A, McGhee SM. Cost-effectiveness of influenza vaccination for elderly people living in the community. Hong Kong medical journal = Xianggang yi xue za zhi. 2009;15 Suppl 6:44-7.

8. Centre for Health Protection. Eligible groups of Government Vaccination Programme (GVP) 2018/19. Available at: https://www.chp.gov.hk/files/pdf/201819_gvp_provisional_table_eng.pdf. 2018.

9. Legislative Council. Seasonal influenza vaccination. Available at: https://www.legco.gov.hk/research-publications/english/essentials-1718ise06-seasonal-influenza-vaccination.htm. 2018.

10. Kan T, Zhang J. Factors influencing seasonal influenza vaccination behaviour among elderly people: a systematic review. Public health. 2018;156:67-78.

11. Thomas RE, Lorenzetti DL. Interventions to increase influenza vaccination rates of those 60 years and older in the community. The Cochrane database of systematic reviews. 2018;5:CD005188.

12. Leung KC, Mui C, Chiu WY, Ng YY, Chen MHY, Ho PH, et al. Impact of patient education on influenza vaccine uptake among community-dwelling elderly: a randomized controlled trial. Health education research. 2017;32(5):455-64.

13. Michie S, Johnston M, Francis J, Hardeman W, Eccles M. From theory to intervention: mapping theoretically derived behavioural determinants to behaviour change techniques. Appl Psychol. 2008;57:660-80.

14. Muellmann S, Forberger S, Mollers T, Broring E, Zeeb H, Pischke CR. Effectiveness of eHealth interventions for the promotion of physical activity in older adults: A systematic review. Preventive medicine. 2018;108:93-110.

15. Prochaska JO, Velicer WF. The transtheoretical model of health behavior change. American journal of health promotion : AJHP. 1997;12(1):38-48.

16. Noar SM, Benac CN, Harris MS. Does tailoring matter? Meta-analytic review of tailored print health behavior change interventions. Psychological bulletin. 2007;133(4):673-93.

17. Lach HW, Everard KM, Highstein G, Brownson CA. Application of the transtheoretical model to health education for older adults. Health promotion practice. 2004;5(1):88-93.

18. Paiva AL, Lipschitz JM, Fernandez AC, Redding CA, Prochaska JO. Evaluation of the acceptability and feasibility of a computer-tailored intervention to increase human papillomavirus vaccination among young adult women. Journal of American college health : J of ACH. 2014;62(1):32-8.

19. Redding CA, Brown-Peterside P, Noar SM, Rossi JS, Koblin BA. One session of TTM-tailored condom use feedback: a pilot study among at-risk women in the Bronx. AIDS Care. 2011;23(1):10-5.

20. Di Noia J, Prochaska JO. Mediating variables in a transtheoretical model dietary intervention program. Health education & behavior : the official publication of the Society for Public Health Education. 2010;37(5):753-62.

21. Kumar R, Rose CP. Triggering effective social support for online groups. ACM Transactions on Interactive Intelligent Systems (TiiS). 2014;3(4):24.

22. Bickmore TW, Schulman D, Sidner C. Automated interventions for multiple health behaviors using conversational agents. Patient education and counseling. 2013;92(2):142-8.

23. Wang H, Zhang QP, Ip M, Lau JT. Social media-based conversational agents for health management and interventions. IEEE. 2018;51(8):26-33.

24. Lau JT, Kim JH, Yang X, Tsui HY. Cross-sectional and longitudinal factors predicting influenza vaccination in Hong Kong Chinese elderly aged 65 and above. The Journal of infection. 2008;56(6):460-8.

25. Del Re AC, Maisel NC, Blodgett JC, Finney JW. Intention-to-treat analyses and missing data approaches in pharmacotherapy trials for alcohol use disorders. BMJ open. 2013;3(11):e003464.
