# Supplementary material for: Chatbot-Delivered Online Intervention to Promote Seasonal Influenza Vaccination During the COVID-19 Pandemic: A Randomized Clinical Trial
Source: JAMA Netw Open. 2023 Sep 11;6(9):e2332568. doi: 10.1001/jamanetworkopen.2023.32568 (PMC10495860; doi:10.1001/jamanetworkopen.2023.32568)
Supplement: Supplement 2. — eAppendix 1. Development of Intervention Materials eAppendix 2. Development and Maintenance of the Intervention System eAppendix 3. Comparing Baseline Characteristics Between Participants Who Completed Month 6 Evaluation and Those Who Were Lost to Follow-Up eAppendix 4. Changes in Stage of Changes Documented by the Chatbot Among Participants Who Had Completed at Least Two Intervention Sessions in the Intervention Group eAppendix 5. Compliance to the Interventions eAppendix 6. Process Evaluation [file jamanetwopen-e2332568-s002.pdf]

Supplemental Online Content

Wang Z, Chan PS, Fang Y, et al. Chatbot-delivered online intervention to promote seasonal influenza vaccination during the COVID-19 pandemic: a randomized clinical trial. *JAMA Netw Open*. 2023;6(9):e2332568.
doi:10.1001/jamanetworkopen.2023.32568

eAppendix 1. Development of Intervention Materials

eAppendix 2. Development and Maintenance of the Intervention System

eAppendix 3. Comparing Baseline Characteristics Between Participants Who Completed Month 6 Evaluation and Those Who Were Lost to Follow-Up

eAppendix 4. Changes in Stage of Changes Documented by the Chatbot Among Participants Who Had Completed at Least Two Intervention Sessions in the intervention Group

eAppendix 5. Compliance to the Interventions

eAppendix 6. Process Evaluation

This supplemental material has been provided by the authors to give readers additional information about their work.

© 2023 Wang Z et al. *JAMA Network Open.*

eAppendix 1. Development of intervention materials

1. Identify facilitators and barriers to receive seasonal influenza vaccination (SIV) among older adults in Hong Kong

Literature review identified seven studies investigating facilitators and barriers to receive SIV among older adults in Hong Kong (Table 1). Perceived benefit of SIV in preventing influenza, belief that influenza would have severe health impact, and receiving recommendation from doctors and family members were facilitators to receive SIV. Barriers to receive SIV included perceived low risk of contracting influenza, concern about side effects of SIV, and not knowing where to receive SIV.

In-depth interviews were performed to further understand older adults' perspective of SIV during the COVID-19 pandemic. Five community-dwelling Chinese-speaking individuals aged ≥65 years were recruited through purposive sampling (2 males and 3 females). Prior to the interview, fieldworkers explained the purposes and nature of the interviews. With verbal informed consent, interviews were conducted through telephone and audio recorded with informants' consent. The interviews lasted for about one hour. We transcribed interviews, kept a codebook to record special data, and transformed the data into categories to identify main themes. Three out of five informants intended to receive SIV for the incoming flu season. The qualitative findings confirmed the facilitators and barriers found in the literature review. In addition, belief that co-infection of seasonal influenza and COVID-19 would lead to severer consequences and death was suggested by two informants as a reason to receive SIV.

2. Generation of health communication messages
A panel consisting of the investigators (public health researchers, experts in behavioral health, health psychologist, and family medicine physician) and two local older adults was formed. Considering the findings of the discussion group and factors found in our previous study, panel members created lists of themes and key health communication messages. Multiple meetings were held to discuss and rank the messages about the relevance and significance. The short-listed messages were tested in another discussion group of five local older adults. The participants were invited to share their opinions on: 1) relevance of these messages to their context, 2) potential influence of these messages on their uptake of seasonal influenza vaccination; 3) practical suggestions for modifying these messages and 4) other important messages which might have been missed. Two independent researchers listened to the tapes and draw out key suggestions. Such suggestions were discussed in a panel meeting in order to finalize the messages.

3. Understand older adults' preference about the format of interventions
The aforementioned in-depth interviews of older adults also explored their preference about the format of the interventions. All informants preferred watching videos covering the health communication messages. There were several reasons to support their choice: 1) a video is more attractive than long text messages, 2) watching a video can help them better digest the information, and 3) difficult to read text on the smartphone due to poor eyesight and/or low literacy level. They also provided some suggestion for the videos and interventions: 1) the video should be short. They might lose interest if the videos were too long. Moreover, they concerned that watching a long video would cost extra money as they had limited data plan, 2) having some peers to share their "real" experience of SIV was more credible and 3) a telephone hotline for enquiry was helpful. We followed their preference and applied audio-

© 2023 Wang Z et al. *JAMA Network Open.*

visual approach to deliver health communication messages. Their suggestions were also taken into consideration when developing the online interventions.

4. Pilot testing of the intervention materials
A pilot study of six eligible participants randomized into the two groups was conducted to test the logistics of the intervention in November 2021. The results of the pilot study showed that the intervention was running smoothly and we did not make any further adjustment to the logistics.

Table 1. Studies investigating facilitators and barriers to receive seasonal influenza vaccination among older adults in Hong Kong

Study	Participants	Uptake rate and associated factors
Lau, 2007 [1]	877 and 1103 respondents aged ≥65 years in two random telephone surveys in 2004 and 2005	**Lifetime SIV uptake**<br>65-69 years: 19.0% (2004) and 38.1% (2005)<br>70-79 years: 26.6% (2004) and 41.1% (2005)<br>≥80 years: 34.6% (2004) and 42.2% (2005)<br>All: 26.6% (2004) and 40.3% (2005)<br>**SIV uptake in the last six months**<br>65-69 years: 16.5% (2004) and 27.4% (2005)<br>70-79 years: 24.9% (2004) and 32.8% (2005)<br>≥80 years: 30.4% (2004) and 35.3% (2005)<br>All: 24.3% (2004) and 31.5% (2005)<br>**Factors associated with SIV uptake**<br>Facilitators:<br>1) Perceived benefit (lack of side effects, SIV is efficacious,<br>2) Perceived severity (severe health impact on oneself if contracting influenza)<br>Barriers<br>1) Perceived barriers (financial difficulties)
Lau, 2008 [2]	886 Chinese elderly aged ≥65 years recruited from random telephone calls	**SIV uptake**<br>25.1% had ever taken up SIV at baseline<br>13% had taken up SIV for the first time during 10-week follow-up period<br>**Factors associated with baseline SIV uptake**<br>Facilitators:<br>1) Know where to go if want to be vaccinated<br>2) Inclined to be vaccinated in the next 12 months if free of charge<br>3) SIV does not have side effects<br>Barriers:<br>1) Had financial difficulties for SIV<br>**Predictors of SIV uptake during follow-up period**<br>Facilitators: have visited old-aged social center
Kwong, 2009 [3]	197 Chinese elderly attending five general outpatient clinics	**SIV uptake:** 64.47% (last 12 months)<br>**Factors associated with SIV uptake**<br>Facilitators:<br>1) Perceived benefits of SIV ("vaccination prevents me from catching influenza", "if I get vaccinated, I will decrease the frequency of medical consultation")

© 2023 Wang Z et al. *JAMA Network Open.*

		2) Cue to action (receive recommendation from doctors and families)<br>Barriers:<br>1) Perceived barriers of SIV ("if I am vaccinated and still get flu, I will not be as sick with it", "the side-effects of SIV interfere with my usual activities", "SIV is painful", "I am scared of needles")<br>**Theoretical framework:** Health Belief Model
Lau, 2009 [4]	816 Chinese elderly aged ≥65 years recruited from social elderly centers	**SIV uptake**: 62.4% (life-time)<br>**Factors associated with SIV uptake**<br>Facilitators:<br>1) Consideration of vaccination in the subsequent years<br>2) Consideration of vaccination if all people aged 65 or above were eligible to receive free vaccination<br>3) Belief that there is a need to receive SIV following SARS and avian influenza<br>4) Receive advice from nursing staff of elderly centers<br>5) Receive advice from medical staff of elderly centers<br>6) Receive advice from family members or friends
Yu, 2014 [5]	306 Chinese elderly having medical risk status of influenza and its serious complication, attending three major general outpatient clinics	**SIV uptake**: 58.5% (lifetime)<br>**Intention to take up SIV in the next year**: 36.3%<br>**Factors associated with intention to take up SIV**:<br>Facilitators:<br>1) Presence of multi-morbidity<br>2) Perceived susceptibility<br>Barriers<br>1) Post-vaccination discomfort<br>**Theoretical framework:** Health Belief Model
Mo, 2015 [6]	1,101 Chinese elderly aged ≥65 years recruited by random telephone calls	**SIV uptake**: 48.5% (lifetime)<br>**Intention to take up SIV**: 49.5% (next 12 months)<br>**Factors associated with SIV uptake/intention to take up SIV**<br>Facilitators:<br>1) Socio-demographics (being female, participation in community centers' activities in the last 12 months)<br>2) History of chronic diseases<br>3) Knowledge related to SIV<br>4) Perceived benefits (perceived efficacy of SIV)<br>5) Cue to action (recommendation from healthcare professionals to receive IV)<br>Barriers:<br>1) Perceived barriers (perceived side effects of SIV)<br>**Theoretical framework:** Health Belief Model
Chan, 2015 [7]	4204 elderly person participated in household survey	**SIV uptake**: 27% (lifetime)<br>**Factors associated with SIV uptake**<br>Facilitators:<br>1) Age ≥70<br>2) With chronic diseases<br>Barriers<br>1) Being male

© 2023 Wang Z et al. *JAMA Network Open.*

		2) Being economically active<br>3) Attained primary education<br>4) Having smoking behaviors

References
[1] Lau JT, Kim JH, Choi KC, Tsui HY, Yang X. Changes in prevalence of influenza vaccination and strength of association of factors predicting influenza vaccination over time --- results of two population-based surveys. Vaccine, 2007; 25: 8279-8289
[2] Lau JT, Kim JH, Yang X, Tsui HY. Cross-sectional and longitudinal factors predicting influenza vaccination in Hong Kong Chinese elderly aged 65 years and above. Journal of Infection, 2008; 56: 460-468
[3] Kwong EW, Lam IO, Chan TM. What factors affect influenza vaccine uptake among community-dwelling older Chinese people in Hong Kong general outpatient clinics? Journal of Clinical Nursing, 2008; 18: 960-971
[4] Lau L, Lau Y, Lau HY. Prevalence and correlates of influenza vaccination among non-institutionalized elderly people: an exploratory cross-sectional survey. International Journal of Nursing Studies, 2009; 46: 768-777
[5] Yu DSF, Low LPL, Lee IFK, Lee DTF, Ng WM. Predicting influenza vaccination intent among at-risk Chinese older adults in Hong Kong. Nursing Research, 2014; 63: 270-277
[6] Mo PKH, Lau JTF. Influenza vaccination uptake and associated factors among elderly population in Hong Kong: the application of the Health Belief Model. Health Education Research, 2015; 30(5): 706-718
[7] Chan DPC, Wong NS, Wong ELY, Cheung AWL, Lee SS. Household characteristics and influenza vaccination uptake in the community-dwelling elderly: a cross-sectional study. Preventive Medicine Reports, 2015; 2: 803-818

© 2023 Wang Z et al. *JAMA Network Open.*

eAppendix 2. Development and maintenance of the intervention system

Development of the intervention system

The architecture of the intervention system was adapted from a mature rule-based Chatbot for smoking cessation [1]. The rule-based Chatbot used a series of pre-defined rules, like a flowchart, to map out human-machine conversation.

The adaption of the rule-based Chatbot for this study was informed by in-depth interviews with older adults. Five community-dwelling Chinese-speaking individuals aged ≥65 years were recruited through purposive sampling (2 males and 3 females). Prior to the interview, fieldworkers explained the purposes and nature of the interviews. With verbal informed consent, interviews were conducted through telephone and audio recorded with informants' consent. The interviews lasted for about one hour. The informants give the following suggestions to adapt the Chatbot.

1) The human-machine interaction should be simple. Most of the older adults wished to obtain information they needed without going through too many rounds of communication with the intervention system. Therefore, we simplified the workflow of the human-machine interactions by asking fewer questions to identify users' status.

2) Using videos to deliver complicated health promotion messages. Many older adults preferred to receive health promotion messages in the format of videos (with Chinese subtitles), as compared to voice/text messages only. There were several reasons to support their choice: 1) a video is more attractive than long text messages, 2) watching a video can help them better digest the information, and 3) difficult to read text on the smartphone due to poor eyesight and/or low literacy level. We followed their preference and used online videos to disseminate health promotion messages, instead of using a fully conversational way.

3) Avoiding typing on the smartphone. Most informants complained input a message/reply by typing the keyboard or handwriting on their smartphone was not easy for them. Therefore, when answering a question raised by the intervention system, they preferred to click a button shown on screen, input a number or letter representing an answer, or use voice messages. All these three options were made available in the present intervention system.

We adopted WhatsApp platform to implement the Chatbot. The Chatbot is integrated with WhatsApp through its public Web API services. Participants' messages are sent to WhatsApp's instant messaging server and to a separated constructed Chatbot system (an administrative system and the Chatbot). The Chatbot system processes a message and sends it back to the WhatsApp instant messaging server. Finally, the users can view the message sent by the Chatbot. The entire process spans less than a second, without sluggish feel for users. The architecture of the Chatbot system is presented in Figure 1.

The intervention system contains three modules:

1) Dialogue management module: The dialogue system records all conversations between users and the Chatbot, so that the system can extract context information such as basic statistics of users' activities and prior interaction between a user and the Chatbot. The natural language processing module analyzes the text content of each message. Then, the message is forwarded to trigger certain actions on the basis of preprogrammed rules. For example, when a user sends a message containing a specific keyword, the Chatbot immediately responds with information that corresponds to that keyword. In addition, the module initiates new conversations on the basis of preprogrammed intervention plan.

2) User management module: This module records detailed information about user conversation. Administrators can link users' WhatsApp number with the Chatbot and review

© 2023 Wang Z et al. *JAMA Network Open.*

the progress of the intervention delivery (e.g., number of completed sessions, disconnection between users and the Chatbot).
3) Multi-media management module: The Chatbot system supports image uploading, sending, and updating through this module to allow image and video exchange.

Pilot testing and refining Chatbot
We purposively recruited 10 community-dwelling Chinese-speaking individuals aged ≥65 years (4 males and 6 females) to use to Chatbot. With informed consent, their conversation with the Chatbot was retrieved and reviewed by the research team. Feedbacks of the users were also collected. All participants believed the Chatbot was easy to use. The research team refined the Chatbot based on results of pilot testing.

Maintenance of the Chatbot
The same professional team maintained the healthy state of the Chatbot during the project period. The interactions between the Chatbot and users were reviewed monthly. The research team identified sections of conversations that leads to users disconnecting with the chat and find out whether there is anything in common between instances. In addition, the team also looked for other areas in which the Chatbot was underperforming. Improvement was made to improve its performance.

Reference
[1] Wang H, Zhang Q, Ip M, Lau JTF. Conversational agents for health management and interventions. Computer, 2018; 51(8): 26-33

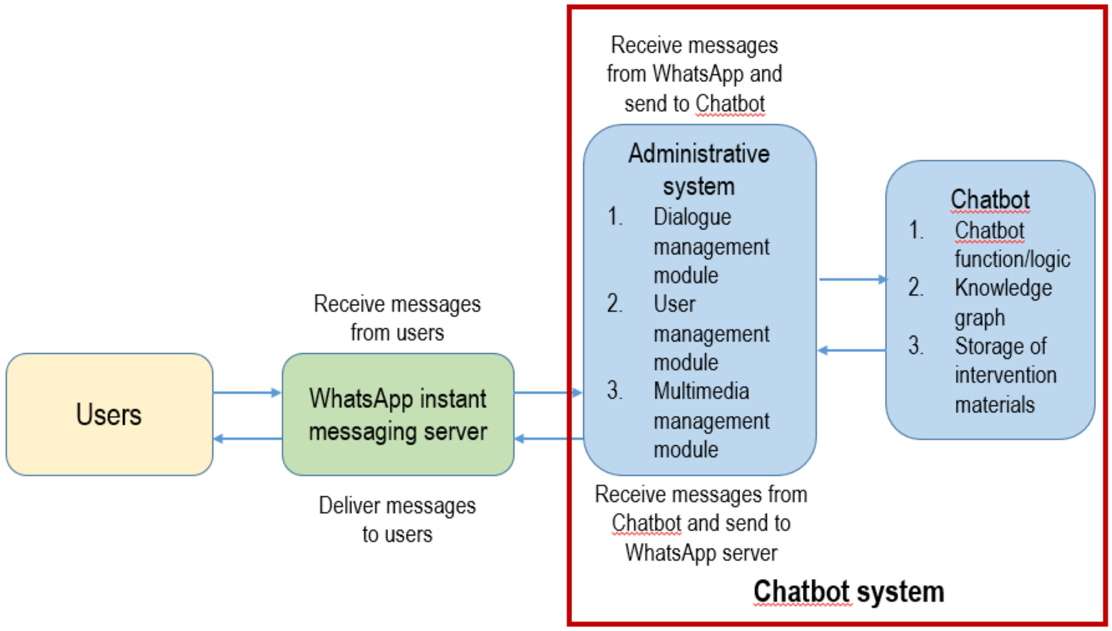

Figure 1: Architecture of the intervention system

© 2023 Wang Z et al. *JAMA Network Open.*

eAppendix 3. Comparing baseline characteristics between participants who completed Month 6 evaluation and those who were lost to follow-up

Table 1 Comparing baseline characteristics between participants who completed Month 6 evaluation and those who were lost to follow-up

Characteristics	Intervention group (n=198)			Control group (n=198)		
	Being followed up (n=174)	Loss-to-follow-up (n=24)	P values	Being followed up (n=165)	Loss-to-follow-up (n=33)	P values
	n (%)	n (%)		n (%)	n (%)	
**Sociodemographic characteristics**						
Age group, year						
65-69	90 (51.7)	14 (58.3)		79 (47.9)	18 (54.5)	
70-74	58 (33.3)	7 (29.2)		60 (36.4)	9 (27.3)	
≥75	26 (14.9)	3 (12.5)	.83	26 (15.8)	6 (18.2)	.61
Sex						
Male	70 (40.2)	11 (45.8)		55 (33.3)	11 (33.3)	
Female	104 (59.8)	13 (54.2)	.60	110 (66.7)	22 (66.7)	1.00
Relationship status						
Currently single	42 (24.1)	5 (20.8)		50 (30.3)	9 (27.3)	
Married or cohabiting with a partner	132 (75.9)	19 (79.2)	.72	115 (69.7)	24 (72.7)	.73
Education level						
Primary or below	73 (42.0)	13 (54.2)		68 (41.2)	10 (30.3)	
Secondary	70 (46.0)	9 (37.5)		79 (47.9)	21 (63.6)	
Tertiary or above	21 (12.1)	2 (8.3)	.52	18 (10.9)	2 (6.1)	.24
Monthly household income, HK$ (US$)						
<20,000 (2580)	127 (73.0)	17 (70.8)		123 (75.0)	27 (81.8)	
≥20,000 (2580)	22 (12.6)	5 (20.8)		22 (13.4)	3 (9.1)	
Refuse to disclose	25 (14.4)	2 (8.3)	.45	19 (11.6)	3 (9.1)	.70
Receiving Comprehensive Social Security Assistance (CSSA)						
No	163 (93.7)	22 (91.7)		148 (89.7)	33 (100.0)	
Yes	11 (6.3)	2 (8.3)	.71	17 (10.3)	0 (0.0)	.054
Living alone						
No	138 (79.3)	20 (83.3)		136 (82.4)	27 (81.8)	

© 2023 Wang Z et al. *JAMA Network Open.*

Yes	36 (20.7)	4 (16.7)	.65	29 (17.6)	6 (18.2)	.93
**Lifestyle and health conditions**						
Smoking in the past year						
No	163 (93.7)	22 (91.7)		153 (92.7)	31 (93.9)	
Yes	11 (6.3)	2 (8.3)	.71	12 (7.3)	2 (6.1)	.80
Binge drinking in the past year						
No	171 (98.3)	23 (95.8)		161 (97.6)	32 (97.0)	
Yes	3 (1.7)	1 (4.2)	.43	4 (2.4)	1 (3.0)	.84
Presence of the following chronic condition, yes						
Hypertension	89 (51.1)	11 (45.8)	.63	70 (42.4)	19 (57.6)	.11
Chronic cardiovascular diseases	18 (10.3)	1 (4.2)	.34	19 (11.5)	4 (12.1)	.92
Chronic lung diseases	4 (2.3)	2 (8.3)	.11	2 (1.2)	0 (0.0)	.53
Chronic liver diseases	5 (2.9)	0 (0.0)	.40	2 (1.2)	1 (3.0)	.44
Chronic kidney diseases	2 (1.1)	0 (0.0)	.60	0 (0.0)	1 (3.0)	.17
Diabetes Mellitus	35 (20.1)	4 (16.7)	.69	30 (18.2)	6 (18.2)	1.00
Any of above	115 (66.1)	12 (50.0)	.12	89 (53.9)	23 (69.7)	.10
History of COVID-19						
No	173 (99.4)	23 (95.8)		162 (98.2)	32 (97.0)	
Yes	1 (0.6)	1 (4.2)	.10	3 (1.8)	1 (3.0)	.65
**Vaccination history**						
History of seasonal influenza vaccination (SIV)						
No	58 (33.3)	15 (62.5)		71 (43.0)	15 (45.5)	
Yes	116 (66.7)	9 (37.5)	.01	94 (57.0)	18 (54.5)	.80
Number of doses of SIV received in the past three years						
0	67 (38.5)	19 (79.2)		77 (46.7)	17 (51.5)	
1	14 (8.0)	0 (0.0)		18 (10.9)	1 (3.0)	
2	19 (10.9)	2 (8.3)		20 (12.1)	7 (21.2)	
3	74 (42.5)	3 (12.5)	.002	50 (30.3)	8 (24.2)	.27
History of pneumococcal vaccination						
No	123 (70.7)	22 (91.7)		121 (73.3)	27 (81.8)	

© 2023 Wang Z et al. *JAMA Network Open.*

Yes	51 (29.3)	2 (8.3)	.03	44 (26.7)	6 (18.2)	.31
Number of doses of COVID-19 vaccination						
0	63 (36.2)	13 (54.2)		63 (38.2)	14 (42.4)	
1	3 (1.7)	0 (0.0)		4 (2.4)	1 (3.0)	
2	108 (62.1)	11 (45.8)	.21	98 (59.4)	18 (54.5)	.87
**Stage of changes**						
Stage of changes related to SIV						
Pre-contemplation stage	53 (30.5)	11 (45.8)		68 (41.2)	16 (48.5)	
Contemplation stage	43 (24.7)	5 (20.8)		31 (18.8)	8 (24.2)	
Preparation stage	78 (44.8)	8 (33.3)	.31	66 (40.0)	9 (27.3)	.38

© 2023 Wang Z et al. *JAMA Network Open.*

eAppendix 4. Changes in stage of changes documented by the Chatbot among participants who had completed at least two intervention sessions in the intervention group

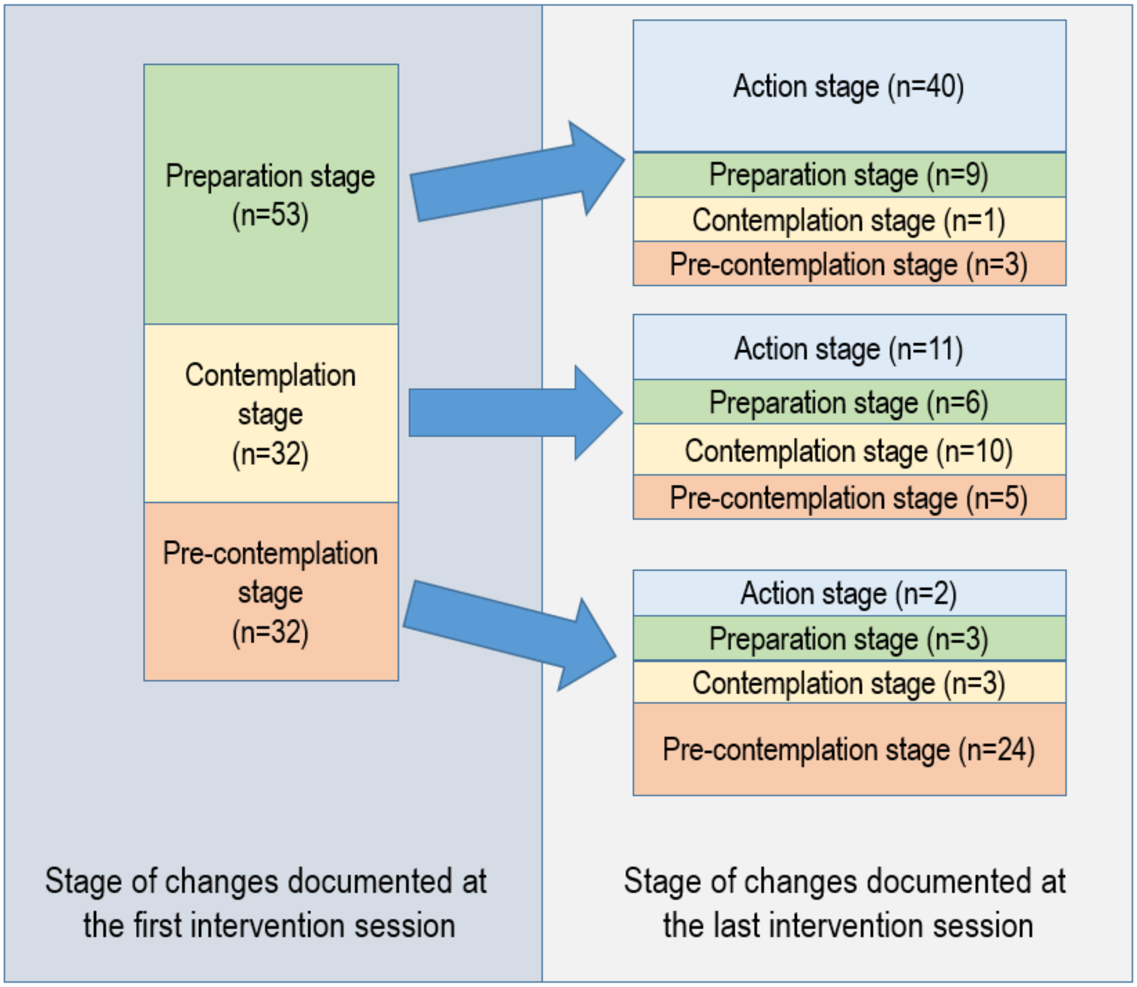

Figure 1. Stages of changes documented by the Chatbot at the first and the last intervention session

Table 1. Changes in the stage of changes among participants who had completed at least two intervention sessions in the intervention group (n=117)

	n (%)
Changes in stage of changes (last intervention session versus first intervention session)	
Stay in the same stage of change	43 (36.8)
Progress to higher stage of change	65 (55.6)
Go back to lower stage of change	9 (7.7)
Score of stage of changes at the first and last intervention session [a], mean (SD)	
First session	2.2 (0.9)
Last session	2.8 (1.3)
P value [b] (last session versus first session)	<0.001

a 1=pre-contemplation stage, 2=contemplation stage, 3=preparation stage, and 4=action stage
b P value was obtained using paired sample t-test

© 2023 Wang Z et al. *JAMA Network Open.*

eAppendix 5. Compliance to the interventions

Table 1 Proportion of participants who interacted with the intervention system and watched online videos at week 0, 2, 4, and 6

	Intervention group	Control group
	n/N (%)	n/N (%)
Answered questions raised by the Chatbot		
Week 0	134/198 (67.7)	121/198 (61.1)
Week 2	114/198 [1] (57.6)	88/198 (44.4)
Week 4	77/165 [2] (46.7)	66 (33.3)
Week 6	46/152 (30.3)	55 (27.8)
Watched online videos		
Week 0	134/198 (67.7)	121/198 (61.1)
Week 2	81/165 [3] (49.1)	88/198 (44.4)
Week 4	64/152 [4] (42.1)	66 (33.3)
Week 6	46/152 (30.3)	55 (27.8)
Engaged in messaging with human research staff from week 0 to 6, yes	15/198 (7.6)	17/198 (8.6)
Number of online videos watched by the participants		
Mean [SD]	1.6 [1.4]	1.6 [1.6]
Median [IQR]	1 [1, 3]	1 [0, 4]

[1] 33 participants reported SIV uptake at week 2, the intervention system would not interact with them at week 4 and 6

[2] 13 participants reported SIV uptake at week 4, the intervention system would not interact with them at week 6

[3] 33 participants reported SIV uptake at week 2 and were not invited to watch online videos at week 2

[4] 13 participants reported SIV uptake at week 4 and were not invited to watch online videos at week 4

Table 2. Associations between stages of change measured at baseline survey and completion of at least one intervention session among participants in the intervention and the control group

	n/N (%)	P values [1]
Intervention group		
Participants who were at pre-contemplation stage at baseline	48/64 (75.0)	
Participants who were at contemplation stage at baseline	36/48 (75.0)	
Participants who were at preparation stage at baseline	69/86 (80.2)	0.68
Control group		
Participants who were at pre-contemplation stage at baseline	40/84 (47.6)	
Participants who were at contemplation stage at baseline	27/39 (69.2)	
Participants who were at preparation stage at baseline	57/75 (76.0)	0.001

[1] P values were obtained from chi-square tests

© 2023 Wang Z et al. *JAMA Network Open.*

eAppendix 6. Process evaluation

Among 124 participants in the intervention group who completed the process evaluation at Month 6, 78.2% of them found it easy to interact with the Chatbot, 78.2% were satisfied with the Chatbot-delivered health promotion, and 82.3% and 56.5% perceived the contents of the online videos were clear and attractive. Majority of them perceived the Chatbot-delivered interventions were helpful to increase their understanding about SIV (77.4%), reduce barriers to receive SIV (82.3%), and enhance self-efficacy (56.5%) and intention to receive SIV (78.2%).

At 339 participants who completed Month 6 follow-up survey, 42.8% had exposed to health communication messages or activities promoting SIV for older adults during the project period (44.8% in the intervention group versus 40.6% in the control group, *P*=.43). The most common channels of health communication was TV (intervention: 31.6% versus control: 27.9%, *P*=.45), followed by pamphlets (intervention: 8.6% versus control: 4.8%, *P*=.17), posters or banners (intervention: 4.6% versus control: 8.5%, *P*=.15), advices made by family doctors (intervention: 2.3% versus control: 4.2%, *P*=.31), or health talk (intervention: 1.7% versus control: 3.6%, *P*=.27). At Month 6, 23.3% of participants received a diagnosis of COVID-19 during the project period (24.1% in the intervention group versus 22.4% in the control group, *P*=.71). There was no between-group difference in the completion of primary COVID-19 vaccination series (intervention: 90.2% versus control: 92.7%, *P*=.41) or uptake of COVID-19 booster doses (intervention: 59.8% versus control: 55.2%, *P*=.39) during the project period.

© 2023 Wang Z et al. *JAMA Network Open.*
