# Supplementary material for: Chatbot-Delivered Online Intervention to Promote Seasonal Influenza Vaccination During the COVID-19 Pandemic: A Randomized Clinical Trial
Source: JAMA Netw Open. 2023 Sep 11;6(9):e2332568. doi: 10.1001/jamanetworkopen.2023.32568 (PMC10495860; doi:10.1001/jamanetworkopen.2023.32568)
Supplement: Supplement 3. — Data Sharing Statement [file jamanetwopen-e2332568-s003.pdf]

Data Sharing Statement

Wang. Chatbot-Delivered Online Intervention to Promote Seasonal Influenza Vaccination During the COVID-19 Pandemic. *JAMA Netw Open*. Published September 11, 2023. doi:10.1001/jamanetworkopen.2023.32568

Data
Data available: No

Additional Information
Explanation for why data not available: The data presented in this study are available from the corresponding author upon request. The data are not publicly available as they contain sensitive personal information.
